# Psychometric properties of the Geriatric Anxiety Inventory (GAI) and its short-form (GAI-SF) in a clinical and non-clinical sample of older adults

**DOI:** 10.1017/S1041610214001586

**Published:** 2014-08-11

**Authors:** Carly Johnco, Ashleigh Knight, Dusanka Tadic, Viviana M. Wuthrich

**Affiliations:** Centre for Emotional Health, Department of Psychology, Macquarie University, Sydney, New South Wales, Australia

**Keywords:** anxiety disorders, anxiety, questionnaires, geriatric, Geriatric Anxiety Inventory, older adult

## Abstract

**Background:**

The Geriatric Anxiety Inventory is a 20-item geriatric-specific measure of anxiety severity. While studies suggest good internal consistency and convergent validity, divergent validity from measures of depression are weak. Clinical cutoffs have been developed that vary across studies due to the small clinical samples used. A six-item short form (GAI-SF) has been developed, and while this scale is promising, the research assessing the psychometrics of this scale is limited.

**Methods:**

This study examined the psychometric properties of GAI and GAI-SF in a large sample of 197 clinical geriatric participants with a comorbid anxiety and unipolar mood disorder, and a non-clinical control sample (*N* = 59).

**Results:**

The internal consistency and convergent validity with other measures of anxiety was adequate for GAI and GAI-SF. Divergent validity from depressive symptoms was good in the clinical sample but weak in the total and non-clinical samples. Divergent validity from cognitive functioning was good in all samples. The one-factor structure was replicated for both measures. Receiver Operating Characteristic analyses indicated that the GAI is more accurate at identifying clinical status than the GAI-SF, although the sensitivity and specificity for the recommended cutoffs was adequate for both measures.

**Conclusions:**

Both GAI and GAI-SF show good psychometric properties for identifying geriatric anxiety. The GAI-SF may be a useful alternative screening measure for identifying anxiety in older adults.

## Introduction

The Geriatric Anxiety Inventory (GAI) was developed as a brief dimensional measure of anxiety symptom severity specifically for use with older people (Pachana *et al.*, 2007). The initial study found adequate psychometric properties in a large sample of normal older adults and a smaller sample of psychogeriatric patients (Pachana *et al.*, 2007). Since this initial study, there has been rapid growth in the number of studies evaluating the psychometric properties of this measure in a variety of older adult settings, including community samples (Byrne *et al.*, 2010), psychogeriatric samples (Cheung, 2007; Boddice *et al.*, 2008), residential aged care settings (Boddice *et al.*, 2008), and in-home care recipients (Diefenbach *et al.*, 2009). It has also been validated in health settings, including in patients with Parkinson's disease (Matheson *et al.*, 2012), patients with chronic obstructive pulmonary disease (COPD; Cheung *et al.*, 2012), and in patients with varied levels of cognitive impairment (Boddice *et al.*, 2008; Byrne *et al.*, 2008; Rozzini *et al.*, 2009).

The GAI is one of the first self-report measures of late-life anxiety that adequately addresses many of the notable limitations of using measures developed with a younger sample in an older adult population. The GAI minimizes the emphasis of somatic symptoms which can be confounded with physical health problems in older adults (Pachana *et al.*, 2007). The GAI has a forced choice response format (agree/disagree) and is scored in a single direction which serves to reduce confusion that can present with reverse scored items and the scaled response formats often used with younger adults. Psychometric and methodological literature on self-report measures given to older adults frequently find that reverse scored items form a separate factor, sometimes referred to as a “confusion factor” (Green *et al.*, 1993; Hazlett-Stevens *et al.*, 2004; Pachana *et al.*, 2007; Boddice *et al.*, 2008; Carlson *et al.*, 2011), as these negatively worded items require an ability to recognize and interpret double negatives and use the reverse ends of a rating scale to make an appropriate response. Using this single direction response format, the GAI has demonstrated a single factor (Pachana *et al.*, 2007; Byrne *et al.*, 2010). The GAI refers to emotional functioning over a past week, allowing for symptom tracking on a weekly routine clinical care schedule. All of these facets make the GAI a desirable measure for use with older population.

While the GAI provides many age-appropriate features for ease of administration with older adults, the psychometric information from the surge of validation studies shows generally good convergent validity, but weak divergent validity from depression measures. The GAI's internal consistency is consistently good, ranging from r = 0.91 to 0.95 (Pachana *et al.*, 2007; Byrne *et al.*, 2008; Matheson *et al.*, 2012). The few studies that have examined test-retest reliability find it to be acceptable (r = 0.91–0.99; Pachana *et al.*, 2007; Matheson *et al.*, 2012), and the original development study found acceptable inter-rater reliability using audio taped responses (Pachana *et al.*, 2007). Convergent validity is also consistently good, with the GAI correlating well with other self-report measures of anxiety. For example, Deifenbach *et al.* (2009) found a positive correlation of GAI with a range of anxiety measures, including the Generalized Anxiety Disorder (GAD) Questionnaire for the Diagnostic and Statistical Manual of Mental Disorders, Fourth Edition (DSM-IV), r = 0.653 (Newman *et al.*, 2002), the Penn State Worry Questionnaire (PSWQ), r = 0.794 (Meyer *et al.*, 1990), the abbreviated version of PSWQ (PSWQ-A), r = 0.795 (Hopko *et al.*, 2003), and the Beck Anxiety Inventory, r = 0.613 (Beck *et al.*, 1988). Similarly, Pachana *et al.* (2007) found a positive correlation between GAI and measures such as the Goldberg Anxiety and Depression Scale – anxiety subscale, r = 0.57 (Goldberg *et al.*, 1988), the State-Trait Anxiety Inventory-State, r = −0.44 (Spielberger *et al.*, 1970), Beck Anxiety Inventory, r = 0.63, and PSWQ, r = 0.70. Convergent validity has also been found using structured clinical interviews, including the Mini-International Neuropsychiatric Interview (MINI; Pachana *et al.*, 2007; Byrne *et al.*, 2010; Cheung *et al.*, 2012; Matheson *et al.*, 2012), Anxiety Disorders Interview Schedule (ADIS-IV; Di Nardo *et al.*, 1994), and Composite International Diagnostic Interview (Boddice *et al.*, 2008).

The GAI is frequently correlated with measures of depression. For example, Andrew and Dulin (2007), Byrne *et al.* (2008), and Deifenbach *et al.* (2009) found moderate to strong correlations with GDS (r = 0.62, 0.79, and 0.67 respectively). It is difficult to interpret the meaning of these correlations because some authors describe the correlation with measures of depression as evidence of poor divergent validity (e.g., Diefenbach *et al.*, 2009), while others conclude that due to high comorbidity between anxiety and depressive symptoms among older adults the correlation indicates adequate convergent validity (e.g., Cheung, 2007; Byrne *et al.*, 2008). Other studies note the correlation but do not prefer this as good or bad (e.g., Andrew and Dulin, 2007). The correlation between measures of anxiety and depression is frequently noted in studies with younger adults, perhaps indicating that these measures assess negative affects in general, rather than anxiety and depression specifically (Feldman, 1993; Stulz and Crits-Christoph, 2010).

The original development paper compared scores on GAI with diagnosed GAD on MINI (Rozzini *et al.*, 2009) and suggested a cutoff of 10/11 out of 20 for identifying likely GAD, and a cutoff of 8/9 out of 20 for identifying any anxiety disorder (Pachana *et al.*, 2007). However, this psychogeriatric sample was very small (*N* = 19) and had only eight participants with a diagnosis of GAD, and 11 with a diagnosis of other anxiety disorders. Subsequent studies have suggested similar cutoffs but have generally used small numbers of patients with anxiety disorders. Diefenbach *et al.* (2009) replicated the original findings suggesting a cutoff of 9 for identifying any anxiety disorder; however, again this was based on a small sample of eight participants with any anxiety disorder diagnosis. In a community sample of females, Byrne *et al.* (2010) suggested a cutoff of 8/9 to predict GAD on MINI, but again only a small number of the sample met the diagnostic criteria (*N* = 8/253). In healthy population, a cutoff of 6/7 was found to be optimum for identifying any anxiety disorder in patients with Parkinson's disease, but again using small number with an anxiety disorder diagnosis (*N* = 16, 28% of the sample; Matheson *et al.*, 2012). A far lower cutoff of 2/3 was suggested to identify patients with any anxiety disorder in a sample with COPD, with 14 (25.5% of the sample) participants meeting the diagnostic criteria (Cheung *et al.*, 2012). Overall, variability in optimal cutoff scores is likely to be an artefact of varied samples and the small number of patients who met criteria for an anxiety disorder. More research in larger clinical samples is needed.

Recently, a short five-item version of GAI was developed (GAI-SF; Byrne and Pachana, 2011); however, there are currently few studies evaluating the psychometric properties of this measure, especially in large samples. The scale was developed in a sample consisting of female participants only, so the applicability to a mixed gender sample has not been established. An optimal cutoff of 2/3 was suggested to identify females with any anxiety disorder; however again, only a small number of participants in the sample had a diagnosed anxiety disorder (*N* = 8, 3.3% of the sample; Byrne and Pachana, 2011). This cutoff had 75% sensitivity, and specificity of 87%, with 86% of participants correctly classified (Byrne and Pachana, 2011). Recently, Gerolimatos *et al.* (2013) examined the psychometric properties of GAI and GAI-SF in 75 nursing home residents and found adequate psychometric properties. This study found moderate correlations between GAI, GAI-SF, and GDS-15, and a good divergent validity from measures of adaptive and executive functioning. Results from this study suggested an optimum cutoff score of 9 for GAI (100% sensitivity and 60% specificity) and a score of 2 for GAI-SF (100% sensitivity and 46.2% specificity) for identifying those with an anxiety disorder diagnosis. Although this study represents one of the first independent studies to evaluate the psychometric properties of GAI-SF, and also the first to examine this in a residential care setting, this, similar to others, consisted of a small sample with a diagnosed anxiety disorder (*N* = 10).

The present study aims to replicate previous findings on psychometric properties of GAI in a large clinical sample of older adults meeting criteria for an anxiety disorder and a non-clinical control sample. This study also aimed to assess the psychometric properties of GAI-SF, given this measure is brief and shows potential for use in screening or epidemiological settings, but has few studies assessing the reliability and validity of this measure. In particular, we were interested in assessing the factor structure, convergent and divergent validity as well and the internal reliability of the short form in comparison with the full version. In addition, this study aims to investigate the sensitivity and specificity of the recommended optimal cutoffs for GAI and GAI-SF to identify those with an anxiety disorder diagnosis in a more robust psychogeriatric sample with comorbid anxiety and depression.

## Method

### Participants

There were a total of 256 community-dwelling participants (161 females, age range = 60–88 years, Mean (M) = 67.51, SD = 5.69). Participants were included from data collected as part of other studies, two using a non-clinical control sample of community-dwelling older adults and the clinical participants being drawn from two randomized control trials for the treatment of late life anxiety and depression (Johnco *et al.*, 2013; Wuthrich and Rapee, 2013). The non-clinical sample (*N* = 59, females = 42, age range = 60–86 years, M = 67.56, SD = 6.20) was recruited from local newspaper advertisements. Participants contacted the researchers and were screened on telephone for clinically significant mental health problems, and those reporting significant mental health problem were excluded. Participants’ scores on the self-report measures of anxiety and depression fell in a normal range.

The clinical sample (*N* = 197, females = 119, age range = 61–88 years, M = 67.50, SD = 5.55) was a treatment-seeking sample recruited through local newspaper advertisements for two randomized controlled trials (RCTs). The Anxiety Disorders Interview Schedule for DSM-IV (ADIS-IV; Di Nardo *et al.*, 1994) was administered by postgraduate psychology students and registered psychologists under the supervision of a clinical psychologist. All clinical participants recruited for the trial met criteria for both DSM-IV anxiety and unipolar mood disorder, with either being primary. In the clinical sample, 58% (*N* = 115) had a primary anxiety disorder (35% GAD, 8.1% social phobia) and 42% (*N* = 82) had a primary unipolar mood disorder (26.9% major depressive disorder, 10.7% dysthymia). During selection for RCT, participants reporting current self-harm, active suicidal ideation, psychosis, or bipolar disorder were excluded from the study as the clinic was ill-equipped to deal with these acute risks. Demographic information for both samples is provided in Table 1.

**Table 1.** Demographic and descriptive information for non-clinical and clinical samples

	non-clinical	clinical	
	(*N* = 59) (%)	(*N* = 197) (%)	*F*
Females	71.2	60.4	2.26
Marital status			0.09
Never married	3.4	4.6	
Married	54.2	47.2	
De facto	3.4	3	
Divorced/separated	20.3	32.9	
Widowed	18.6	12.2	
Country of birth			1.97
Australia	69.5	66.0	
UK	18.6	10.6	
Other	11.9	23.4	
Highest level of qualification			1.74
Primary school	0	3.6	
Secondary school	20.3	22.8	
Certificate/diploma	30.5	27.9	
Bachelors degree	32.2	19.3	
Postgraduate degree	15.3	14.7	
Other	1.7	11.7	
Employment status			0.17
Employed full-time	5.1	6.6	
Semi-retired	35.6	20.3	
Retired	55.9	67.0	
Unable to work due to illness/injury	0	2	
Unemployed	3.4	3	
Gross income ($AUD)			1.57
<$15,599	16.7	22.1	
$15,600–41,599	37.0	46.3	
$41,600–83,199	33.3	21.1	
$83,200–134,199	13.0	6.8	
135,200+	0	3.7	
	M (SD)	M (SD)	t
GAI	0.58 (1.32)	11.08 (4.86)	−27.17*
GAI-SF	0.17 (0.62)	3.46 (1.48)	−27.78*
GDS	2.30 (2.83)	17.28 (5.53)	−27.20*
PSWQ-A	13.49 (5.40)	25.15 (7.59)	−10.65*

Note: *p < 0.05.

### Measures

*Addenbrooke Cognitive Examination – Revised* (ACE-R; Mioshi *et al.*, 2006): The ACE-R is a screening instrument designed to assess an individual's cognitive level, and has demonstrated good internal consistency and convergent validity in an older adult sample (Mioshi *et al.*, 2006). This measure was administered to participants by a trained postgraduate psychology student. Results suggest that this sample was cognitively intact with only 2.1% of the sample scoring in the range indicative of cognitive impairment (<82/100; M = 92.71, SD = 4.80). This measure consists of five subscales assessing various aspects of cognitive functioning, and the attention/orientation, memory, fluency, language, and visuospatial subscales are combined to provide an overall total cognitive level score. The attention/orientation, language, and visuospatial subscales were used to assess divergent validity, given they are unlikely to be influenced by anxiety or depression.

*Anxiety Disorders Interview Schedule* (Di Nardo *et al.*, 1994): The ADIS is a semi-structured interview for diagnosing anxiety and related disorders according to DSM-IV criteria. Trained postgraduate psychology students, who received regular supervision, administered it. The interview is designed to support clinicians in determining the presence and severity of disorders using a rating scale of 0–8, where ratings of 4 and above are considered of clinical severity. The ADIS-IV was administered in full to the clinical sample only.

*Geriatric Anxiety Inventory* (Pachana *et al.*, 2007): The GAI is a 20-item measure of anxiety symptoms severity developed for older adults. It has been shown to have adequate internal consistency, test-retest reliability, and concurrent validity (Pachana *et al.*, 2007). Internal consistency was good for the non-clinical (α = 0.73) and clinical samples (α = 0.85) in the current sample.

*Geriatric Anxiety Inventory – Short Form* (Byrne and Pachana, 2011): The GAI-SF is an abbreviated version of GAI comprising only five of the original items. The GAI-SF has good internal consistency, convergent and divergent validity, and was highly correlated with the original GAI (Pachana *et al.*, 2007). In the current study, the internal consistency for the non-clinical sample was adequate (α = 0.71) but marginal for the clinical sample (α = 0.58). The scores from GAI-SF were extracted in the same manner as Byrne and Pachana (2011) from the GAI scores as opposed to the two tests being administered separately.

*Geriatric Depression Scale* (Yesavage *et al.*, 1983): The GDS is a 30-item self-report measure aimed to determine the severity of depressive symptoms in older adults. It has high internal consistency, reliability, sensitivity, and specificity (Yesavage *et al.*, 1983; Kieffer and Reese, 2002; Jongenelis *et al.*, 2005). Internal reliability was good for both non-clinical (α = 0.79) and clinical samples (α = 0.84) in the current study.

*Penn State Worry Questionnaire – Abbreviated* (Hopko *et al.*, 2003): The PSWQ-A is an abbreviated version of PSWQ (Meyer *et al.*, 1990), which contains eight of the original items and assesses worry severity. The PSWQ-A has been shown to be highly correlated with the original measure, have good internal consistency, and convergent-divergent validity in older adult samples (Hopko *et al.*, 2003). In our sample, internal consistency was good for both non-clinical (α = 0.82) and clinical samples (α = 9.90).

### Procedure

The Macquarie University Human Ethics Committee granted ethnic approval for all studies, and participants provided written consent. The clinical participants completed the measures as part of an initial assessment prior to treatment for treatment study. The non-clinical participants completed the measures as part of other studies. Analyses were conducted using SPSS version 17 and STATA version 12. 1 (StataCorp. 2011).

## Results

Group differences on demographic characteristics were examined using one-way ANOVA, and no significant differences were found (see Table 1). Independent sample t-tests were conducted to compare group differences on cognitive (ACE-R total score) and symptom measures (GAI, GAI-SF, GDS, and PSWQ-A) and indicated that the clinical sample scored significantly higher on all symptom measures (see Table 1). Missing data were excluded in all analyses in this paper.

Replicating previous studies, the internal reliability was good for GAI in the total sample (α = 0.926), clinical sample (α = 0.854), and non-clinical sample (α = 0.714). The internal reliability for GAI-SF was good for the total sample (α = 0.840) and the non-clinical sample (α = 0.718), and acceptable for the clinical sample (α = 0.669).

There was moderate convergent validity of GAI and GAI-SF with another measure of anxiety (PSWQ-A) in the total sample (r = 0.787, p < 0.001 and r = 0.793, p < 0.001), clinical sample (r = 0.637, p < 0.001 and r = 0.641, p < 0.001 respectively), and non-clinical sample (r = 0.603, p < 0.001 and r = 0.563, p < 0.001). The GAI and GAI-SF were significantly correlated in the total sample (r = 0.934, p < 0.001), clinical sample (r = 0.865, p < 0.001) and non-clinical sample (r = 0.893, p < 0.001).

There was some evidence of divergent validity from a measure of geriatric depression (GDS) in the clinical sample with the GAI showing a weaker correlation with the GDS compared with the PSWQ-A (r = 0.477, p = 0.001; z = 2.539, p = 0.011). Similarly, the GAI-SF showed a weaker correlation with GDS compared with PSWQ-A (r = 0.372, p = 0.001; z = 4.092, p < 0.001), suggesting good divergent validity in the clinical sample. The correlations between GAI and GDS, and GAI-SF and GDS, were not significantly different in magnitude in relation to PSWQ-A in the total sample (r = 0.773, p = 0.001; z = 0.52, p = 0.603 and r = 0.737, p = 0.001; z = 2.00, p = 0.05 respectively), suggesting poor divergent validity in the total sample. Similarly, there was no significant difference between these relationships in the non-clinical sample (r = 0.656, p < 0.001; z = −0.554, p = 0.58 and r = 0.520, p < 0.001; z = 0.401, p = 0.689), suggesting weak divergent validity from measures of depression. Divergent validity from measures of cognitive functioning was assessed using the attention/orientation, language and visuospatial subscales from the ACE-R, given that these subscales are unlikely to be negatively influenced by anxiety and depression (compared with the fluency and memory subscales that are commonly found to be impaired in those with anxiety and depressive disorders). There was good divergent validity for GAI and GAI-SF from the attention/orientation subscale in total, clinical, and non-clinical samples (r = −0.048, 0.077, and 0.052 respectively, and all p-values were non-significant) as well as from the language subscale (r = −0.063, 0.066, and −0.018 respectively, all p-values were non-significant) and visuospatial subscale (r = −0.118, −0.074, and 0.73 respectively, all p-values were non-significant).

A factor analysis using principal axis factoring was conducted for total and clinical samples only due to limited variability in the GAI and GAI-SF scores in the non-clinical sample that prohibited separate analysis. In the clinical sample, the Kaiser–Meyer–Olkin Measure of Sampling Adequacy = 0.833 suggested that the sample was large enough for analysis, and inter-correlations between items were adequate (Bartlett's Test of Sphericity, χ^2^ (190) = 1004.286, p < 0. 001). Examination of eigenvalues and scree plot suggest a one-factor solution for the clinical sample that explained 27.07% of variance. Similarly, a one-factor solution was found for the total sample (Kaiser–Meyer–Olkin measure = 0.929, Bartlett's Test of Sphericity, χ^2^ = 2356.326, p < 0.001) that explained 42.06% of variance. Factor loadings are shown in Table 2. For the GAI-SF, the factor analysis for both clinical (Kaiser–Meyer–Olkin measure = 0.753, Bartlett's Test of Sphericity, χ^2^ = 133.320, p < 0.001) and total samples (Kaiser–Meyer–Olkin measure = 0.845, Bartlett's Test of Sphericity, χ^2^ = 453.275, p < 0.001) suggested a one-factor solution that explained 43.90% and 61.28% of variance in clinical and total samples respectively. Factor loadings are shown in Table 3.

**Table 2.** Component matrix of GAI for total sample

item	clinical sample	total sample
Q1	0.610	0.809
Q2	0.370	0.542
Q3	0.558	0.644
Q4	0.403	0.625
Q5	0.586	0.715
Q6	0.599	0.682
Q7	0.504	0.532
Q8	0.518	0.712
Q9	0.548	0.655
Q10	0.643	0.735
Q11	0.500	0.734
Q12	0.382	0.435
Q13	0.488	0.588
Q14	0.447	0.571
Q15	0.501	0.574
Q16	0.558	0.767
Q17	0.557	0.660
Q18	0.482	0.533
Q19	0.526	0.607
Q20	0.528	0.721

**Table 3.** Component matrix of GAI-SF for total sample

item	clinical sample	total sample
Q1	0.760	0.870
Q6	0.638	0.710
Q8	0.702	0.811
Q10	0.672	0.752
Q11	0.516	0.763

**Figure 1.**
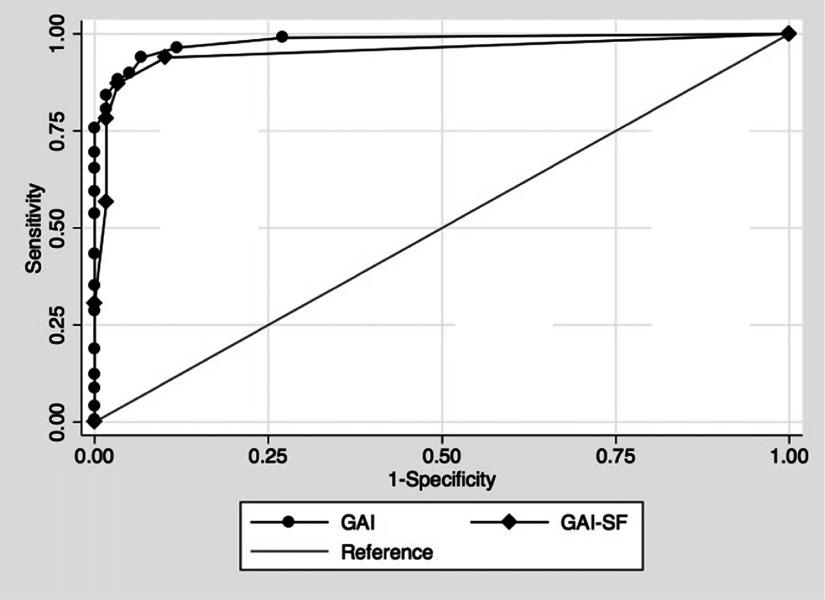
ROC analysis for GAI and GAI-SF using clinical and non-clinical samples *Note:* GAI = Geriatric Anxiety Inventory; GAI-SF = Geriatric Anxiety Inventory – Short Form; ROC = Receiver Operating Characteristics.

Finally, Receiver Operating Characteristic (ROC) analyses were conducted in STATA version 12 to determine the sensitivity and specificity of GAI and GAI-SF in correctly identifying the clinical sample from the non-clinical sample. Test's accuracy is determined by the area under the ROC curve (AUC), where an area of 1 indicates perfect accuracy and 0.5 suggests no greater than chance (see Figure 1). For GAI, the AUC = 0.981 (SE = 0.007, p < 0. 001, 95% CI = 0.967–0.995. The recommended cutoff of 8/9 had 69.5% sensitivity, 100% specificity, and κ = 0.513, p < 0.001. For GAI-SF, the AUC = 0.954 (SE = 0.01, p < 0.001, 95% CI = 0.928–0.980). The recommended cutoff of 2/3 had a sensitivity = 78.14%, specificity = 98.3%, κ = 0.612; p < 0.001. There was a significant difference between GAI and GAI-SF (χ^2^ (1) = 9.52, p = 0.002), indicating that the full GAI was significantly better at identifying those in the clinical group.

## Discussion

This study aimed to examine the psychometric properties of GAI and GAI-SF in clinical and non-clinical samples. We examined the factor structure, convergent and divergent validity as well and the internal reliability of the short form in comparison with the full version. We also investigated the sensitivity and specificity of the recommended optimal cutoffs for GAI and GAI-SF to identify those with a diagnosis of anxiety disorder in a psychogeriatric sample with comorbid anxiety and depression. Focusing on GAI first, our findings support previous studies in terms of good internal consistency for GAI, similar to Pachana *et al.* (2007) and Byrne *et al.* (2010), and good convergent validity between GAI and PSWQ-A among all samples, similar to Diefenbach *et al.* (2009). Consistent with previous studies, scores on GAI were significantly related to clinical status. These findings are consistent with previous work suggesting good convergent validity with other self-report measures of anxiety (Pachana *et al.*, 2007; Byrne *et al.*, 2008; Diefenbach *et al.*, 2009).

For GAI-SF, the internal consistency was adequate in total, non-clinical, and clinical samples, similar to previous findings (Gerolimatos *et al.*, 2013), in a sample of nursing home residents (α = 0.73). Convergent validity with self-report measures was good for total and non-clinical samples, consistent with the development paper (Byrne and Pachana, 2011). A one-factor solution was suggested for both GAI and GAI-SF.

Similar to previous studies, GAI and GAI-SF were significantly correlated with GDS in all samples. While previous studies have been inconsistent with their reporting of this relationship as either evidence of good convergent validity (given the high rates of comorbidity in late-life anxiety and depression) or poor divergent validity (given the poor differentiation), we would suggest that this is an evidence of poor divergent validity. The correlation between GAI and GDS in the clinical sample was significantly weaker than the relationship between GAI and PSWQ-A, suggesting adequate divergent validity in the clinical sample. However, the magnitude of correlations was similar in total and non-clinical samples, suggesting poorer divergent validity from depressive symptoms in total and non-clinical samples. The GAI-SF experienced similar issues with GAI for divergent validity: better divergent validity in the clinical sample, and poorer divergent validity from depression in total and non-clinical samples. The poor divergent validity from GDS in the non-clinical sample is likely a consequence of overlap of anxiety and depression construct more generally, and has been shown to be a common problem with other measures of anxiety, for example, the PSWQ (Hopko *et al*, 2003). Divergent validity from measures of cognitive functioning was good in all the samples.

Both GAI and GAI-SF demonstrated good predictive validity for clinical status; however, the ROC analysis suggested that the full GAI was significantly better at identifying those in the clinical group. Previous studies examining cutoff scores have used very small numbers of patients with a diagnosed anxiety disorder (Pachana *et al.*, 2007; Diefenbach *et al.*, 2009; Byrne *et al.*, 2010; Cheung *et al.*, 2012; Matheson *et al.*, 2012). This is the first study to include a large sample of older adults with a diagnosed anxiety disorder, but replicates previous findings. The ROC analysis suggested adequate sensitivity and specificity using the recommended cutoff of 8/9 for GAI for identifying people with an anxiety disorder, resulting in a sensitivity of 69.5% and specificity of 100%. Similarly, the recommended cutoff of 2/3 for GAI-SF was adequate with 78.14% sensitivity and 98.3% specificity. This study adds to the emerging body of evidence supporting the utility of GAI-SF as a valid screening measure for anxiety disorders in older adults.

We acknowledge potential limitations of this study, including that all clinical participants had a comorbid depressive disorder diagnosis that may have escalated the divergent validity relationship with depression measures. However, given that previous studies have found a significant relationship with measures of depression, and that the weakest relationship was among the clinical sample, we would suggest that the comorbidity of our sample did not unduly influence results. Similarly, the non-clinical sample in this study reported minimal levels of anxiety, with only 10.2% of the sample participants endorsing any anxiety symptoms on GAI-SF and 27.1% endorsing any symptoms on GAI. This may have created a floor effect for ROC analyses, whereby endorsing almost any symptoms on GAI would likely indicate clinical status. Although the present sample ranged in age from 60 to 88 years, the mean age for the total sample indicated that it was a relatively young older adult sample and the results warrant replication in an older sample. A final limitation is that we extracted the GAI-SF scores from the full GAI, as done in Byrne and Pachana (2011). While this reduces potential error caused by administering the scale twice, it is important that further comparisons are made when the two scales are administered separately.

This study is one of the first to examine the psychometric properties of GAI and GAI-SF in a large clinical sample. Results support the existing research that GAI is adequate for detecting anxiety in community-dwelling clinically disordered and non-clinical older adults. Further, our results indicated that GAI-SF is also an adequate measure of anxiety. Although the ROC results indicated that GAI is more accurate at identifying clinical status than the short version, the short version may be useful for screening purposes in primary care or epidemiological settings. Replication of these findings in independent studies using large numbers of clinical participants is needed.

## Conflict of interest

None

## Description of authors’ roles

C. Johnco and V. Wuthrich designed the study, collected and analyzed the data, and wrote the manuscript. A. Knight was responsible for assisting with the statistical analysis of the paper and manuscript preparation. D. Tadic was responsible for collecting the data and revising the paper.
